# Artificial Intelligence in Diabetic Kidney Disease Research: Bibliometric Analysis From 2006 to 2024

**DOI:** 10.2196/72616

**Published:** 2026-01-09

**Authors:** Xingyuan Li, Liming Xiao, Fenghao Yang, Fang Liu

**Affiliations:** 1 Department of Nephrology, West China Hospital, Sichuan University, 37 Guoxue Alley, Wuhou District, Chengdu, Sichuan, 610041, China, 86 13880602361

**Keywords:** artificial intelligence, diabetic kidney disease, bibliometric analysis, clinical validation, explainable AI, global collaboration

## Abstract

**Background:**

Diabetic kidney disease (DKD) is a major complication of diabetes and the leading cause of end-stage renal disease globally. Artificial intelligence (AI) technologies have shown increasing potential in DKD research for early detection, risk prediction, and disease management. However, the landscape of AI applications in this field remains incompletely mapped, especially in terms of collaboration networks, thematic evolution, and clinical translation.

**Objective:**

This study aims to perform a comprehensive bibliometric and translational analysis of AI-related DKD research published between 2006 and 2024, identifying publication trends, research hotspots, key contributors, collaboration patterns, and the extent of clinical validation and explainability.

**Methods:**

A systematic search of the Web of Science Core Collection was conducted to identify English-language original articles applying AI technologies to DKD. Articles were screened following PRISMA (Preferred Reporting Items for Systematic Reviews and Meta-Analyses) 2020 guidelines. Bibliometric visualization was performed using CiteSpace and VOSviewer to assess coauthorship, institutional and country collaboration, keyword evolution, and citation bursts. A qualitative review was conducted to evaluate clinical validation, model explainability, and real-world implementation.

**Results:**

Out of 1158 retrieved records, 384 studies met the inclusion criteria. Global publications on AI in DKD increased rapidly after 2019. China led in publication volume, followed by the United States, India, and Iran. Keyword analysis showed a thematic transition from early biomarker and proteomic research to deep learning, clinical prediction models, and management tools. Despite methodological advances, few studies included external validation or explainability frameworks. Notable translational efforts included DeepMind’s acute kidney injury predictor and a chronic kidney disease prediction model developed by Sumit, yet widespread real-world integration remains limited.

**Conclusions:**

AI research in DKD has grown substantially over the past 2 decades, with expanding international collaboration and diversification of research themes. However, challenges persist in clinical applicability, model transparency, and global inclusivity. Future research should prioritize explainable AI, multicenter validation, and integration into clinical workflows to support effective translation of AI innovations into DKD care.

## Introduction

Diabetic kidney disease (DKD) is the most prevalent microvascular complication of diabetes mellitus and a leading cause of end-stage renal disease globally, accounting for a substantial proportion of dialysis and transplantation burdens worldwide [1]. The pathophysiological progression of DKD is complex, often involving chronic hyperglycemia-induced glomerular injury, hemodynamic changes, inflammation, and fibrosis. Early-stage DKD is typically asymptomatic, and by the time clinical markers such as proteinuria or a decline in glomerular filtration rate become apparent, irreversible kidney damage may have already occurred [2]. Therefore, early detection and individualized risk stratification are essential for improving patient outcomes and alleviating long-term health care burdens.

In this context, artificial intelligence (AI) has emerged as a transformative approach in biomedical research and clinical practice. With capabilities in data-driven pattern recognition, predictive modeling, and real-time decision support, AI techniques—including machine learning, deep learning, and neural networks—have been increasingly explored to address key challenges in DKD research and management [3,4]. Applications range from biomarker discovery and disease classification to risk modeling and personalized treatment optimization. Despite the growing enthusiasm for AI, there is wide variability in the methodological rigor, clinical applicability, and translational maturity of these studies.

While several narrative and systematic reviews have highlighted specific AI models used in nephrology, there remains a lack of comprehensive evaluation of how the field has evolved thematically over time, which countries and institutions are leading its development, how collaborative efforts are shaping knowledge production, and to what extent the proposed AI solutions are being validated and implemented in real-world clinical settings. Moreover, important dimensions such as model explainability, equity in global research representation, and translational readiness are often underexamined.

This study aims to address these gaps by conducting a bibliometric and translational landscape analysis of AI-related DKD research published from 2006 to 2024. By integrating quantitative bibliometric mapping with qualitative evaluation of translational attributes—including clinical validation, model transparency, and implementation potential—we aim to provide a comprehensive overview of this rapidly evolving field and offer insights to inform future research, clinical integration, and policy development.

## Methods

### Literature Search and Eligibility Criteria

A systematic literature search was conducted using the Web of Science Core Collection to identify studies related to the application of AI in DKD from January 1, 2006, to April 30, 2024. The search strategy included combinations of terms for DKD (“diabetic kidney disease,” “diabetic nephropathy,” “DKD,” or “DN”) and AI (“artificial intelligence,” “machine learning,” “deep learning,” or “neural network”). Only English-language articles were considered. The search was limited to original research articles involving human-related data, excluding reviews, editorials, letters, conference abstracts, and purely experimental or theoretical reports without clinical relevance.

Eligible articles were those that applied AI techniques to DKD in a clinical, translational, or predictive context. Studies that involved image processing, signal detection, or statistical models unrelated to DKD-specific diagnostic or prognostic tasks were excluded. To ensure the reliability of inclusion, 2 reviewers (XL and FY) independently screened titles and abstracts for relevance, followed by full-text assessment. Discrepancies were resolved by consensus or consultation with a third reviewer (LX).

### Bibliometric Mapping and Analysis Tools

Bibliometric data were exported from the Web of Science platform (Multimedia Appendix 1) and analyzed using CiteSpace (v6.1.R6) and VOSviewer (v1.6.18; Leiden University's Centre for Science and Technology Studies; Multimedia Appendix 2). These tools enabled visualization and quantification of publication trends, author and institutional productivity, international collaboration networks, and thematic keyword clusters. CiteSpace was used to generate timeline visualizations and detect emergent research topics through keyword burst detection. VOSviewer was applied to construct network maps illustrating coauthorship patterns and co-occurrence frequencies. Centrality scores and citation frequencies were used to identify influential authors, institutions, and countries within the research landscape.

### Translational and Thematic Evaluation

In addition to bibliometric analysis, a qualitative assessment was performed to evaluate the translational significance of the included studies. This review focused on identifying whether AI models were externally validated or tested across different cohorts, whether explainable AI methods were incorporated, and whether any studies reported or discussed clinical integration or real-world implementation. Studies that mentioned the use of interpretability frameworks such as SHAP (Shapley Additive Explanations) or LIME (Local Interpretable Model-Agnostic Explanations) were noted. The presence of multicenter datasets, ethnically diverse populations, or cross-national data integration was also considered as indicators of generalizability and applicability. This dual approach—combining quantitative mapping with thematic content analysis—allowed for a multidimensional perspective on both the scientific growth and translational depth of AI research in DKD.

### Ethical Considerations

This study involved no human participants, animals, or patient data, and therefore did not require ethical approval. The data used were retrieved from publicly available bibliographic databases and do not involve any sensitive or identifiable personal information.

## Results

### Study Selection

A total of 1158 records were initially identified following the PRISMA (Preferred Reporting Items for Systematic Reviews and Meta-Analyses) 2020 framework. After the removal of 0 duplicates, 1158 records were screened based on their titles and abstracts. Of these, 251 records were excluded as irrelevant. The remaining 907 full-text articles were assessed for eligibility, resulting in 384 articles included in the quantitative synthesis, and an additional 78 articles included in the qualitative thematic review. Ultimately, these articles were included in the subsequent bibliometric and qualitative synthesis.

### Publication Growth Over Time

**Figure 1.**
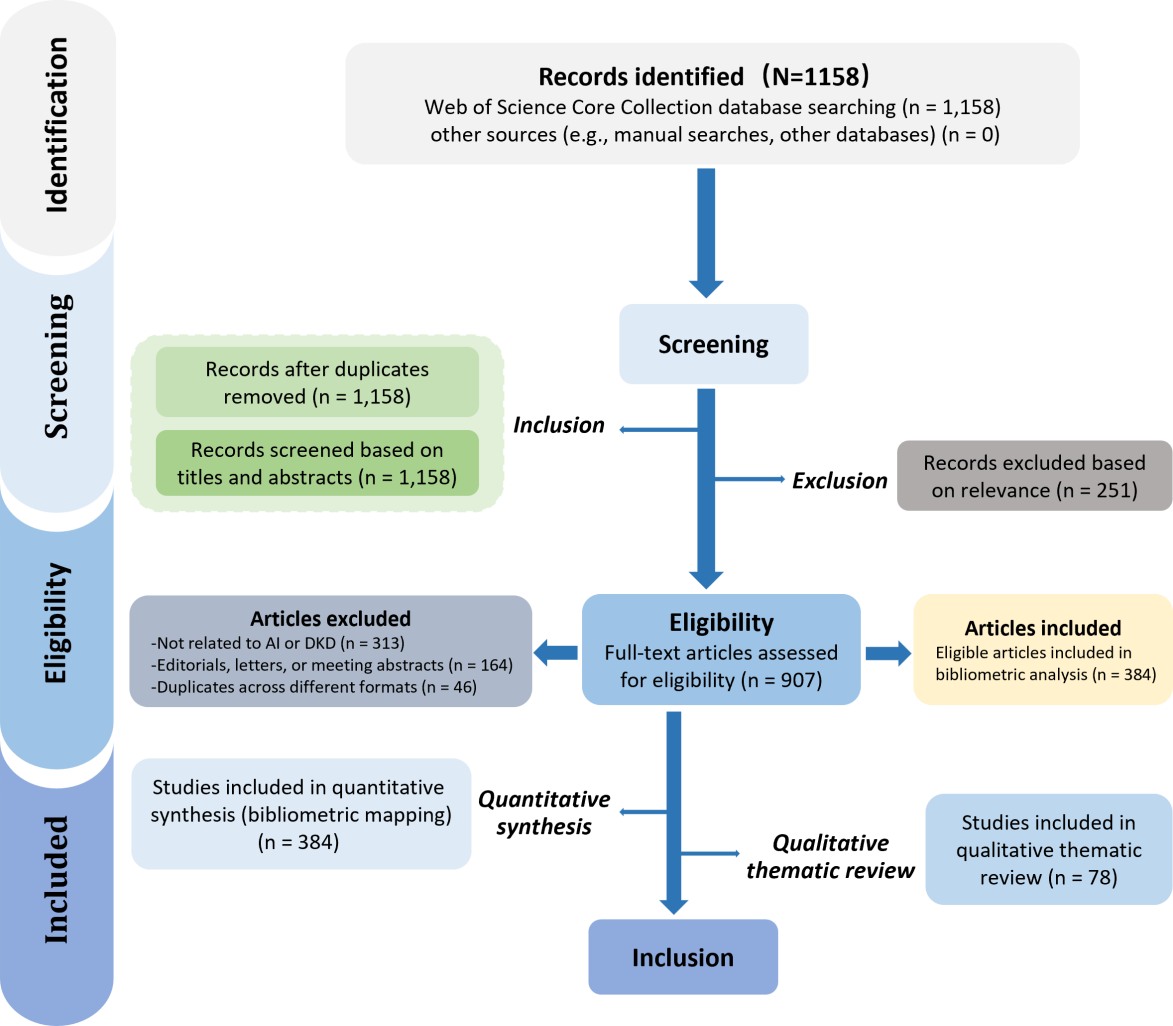
PRISMA (Preferred Reporting Items for Systematic Reviews and Meta-Analyses) 2020 flow diagram for literature screening. AI: artificial intelligence; DKD: diabetic kidney disease.

**Figure 2.**
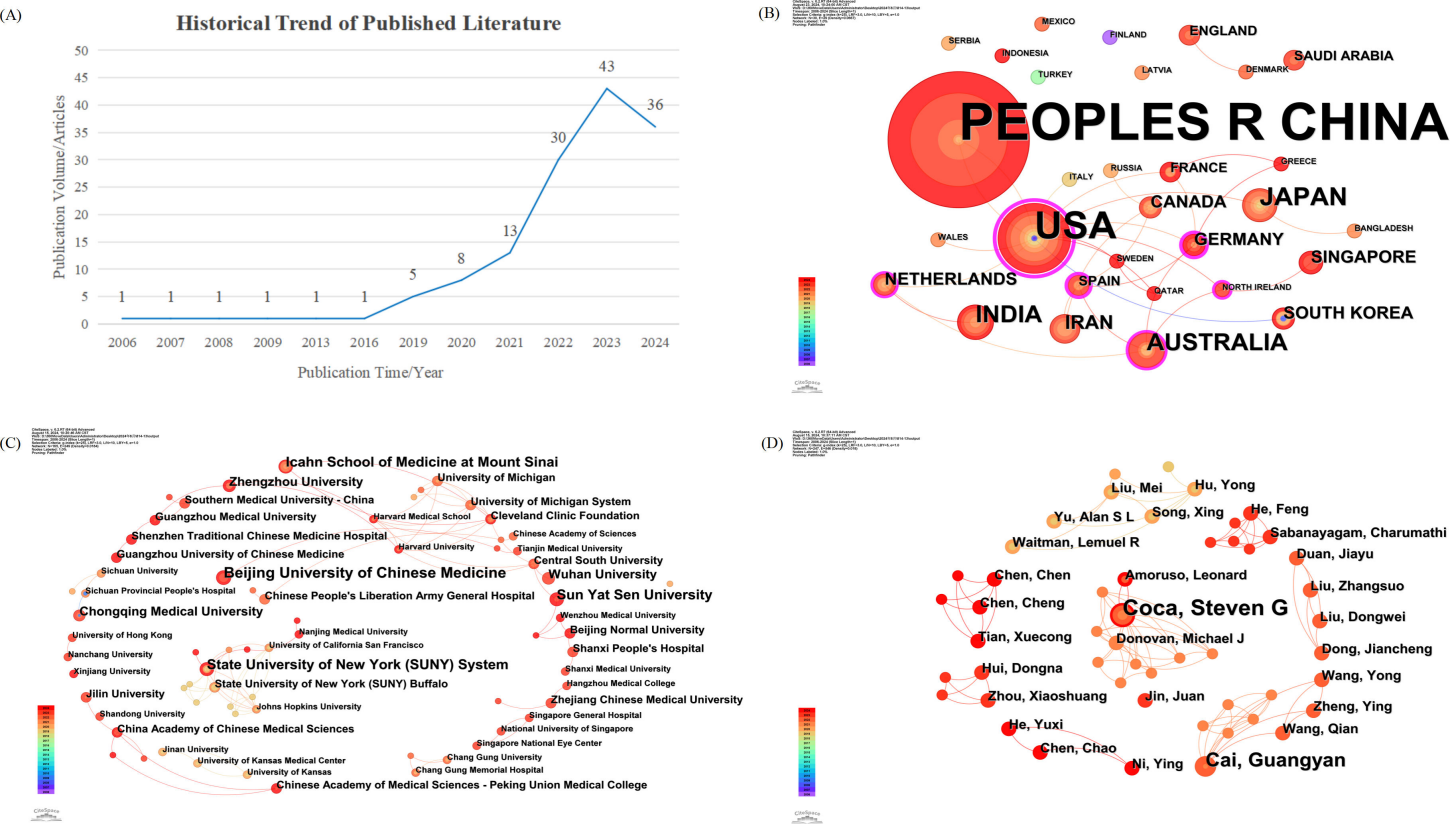
Analysis of the publication trends in artificial intelligence research on diabetic kidney disease from 2006 to 2024: (A) timeline of annual publications, (B) co-occurrence network of research countries, (C) co-occurrence network of research institutions, and (D) co-occurrence network of authors.

The global volume of publications related to AI in DKD remained low and relatively stagnant between 2006 and 2016. A notable increase in research output began in 2019, followed by a rapid rise during the years 2022 to 2024 (Figure 1). This pattern reflects the growing integration of AI techniques into biomedical research and the rising urgency of addressing DKD in the context of the global diabetes epidemic. The sharp upward trend in recent years suggests an increasing recognition of AI as a valuable tool for advancing DKD risk prediction, diagnosis, and management (Figure 2A).

### Geographic and Institutional Contributions

China emerged as the leading contributor in terms of publication volume, accounting for nearly half of all included studies. Key institutions such as Beijing University of Chinese Medicine, Sun Yat-sen University, and Central South University were among the most prolific. The United States ranked second, with prominent contributions from institutions such as the Icahn School of Medicine at Mount Sinai. India, Iran, and Australia also made notable contributions, reflecting a broader international interest in the intersection of AI and nephrology. Collaboration patterns showed that high-output countries often published independently, although intercontinental partnerships—particularly between East Asia, North America, and parts of Europe—have been increasing in frequency and visibility (Figure 2B-D).

### Keyword Evolution and Research Hotspots

**Figure 3.**
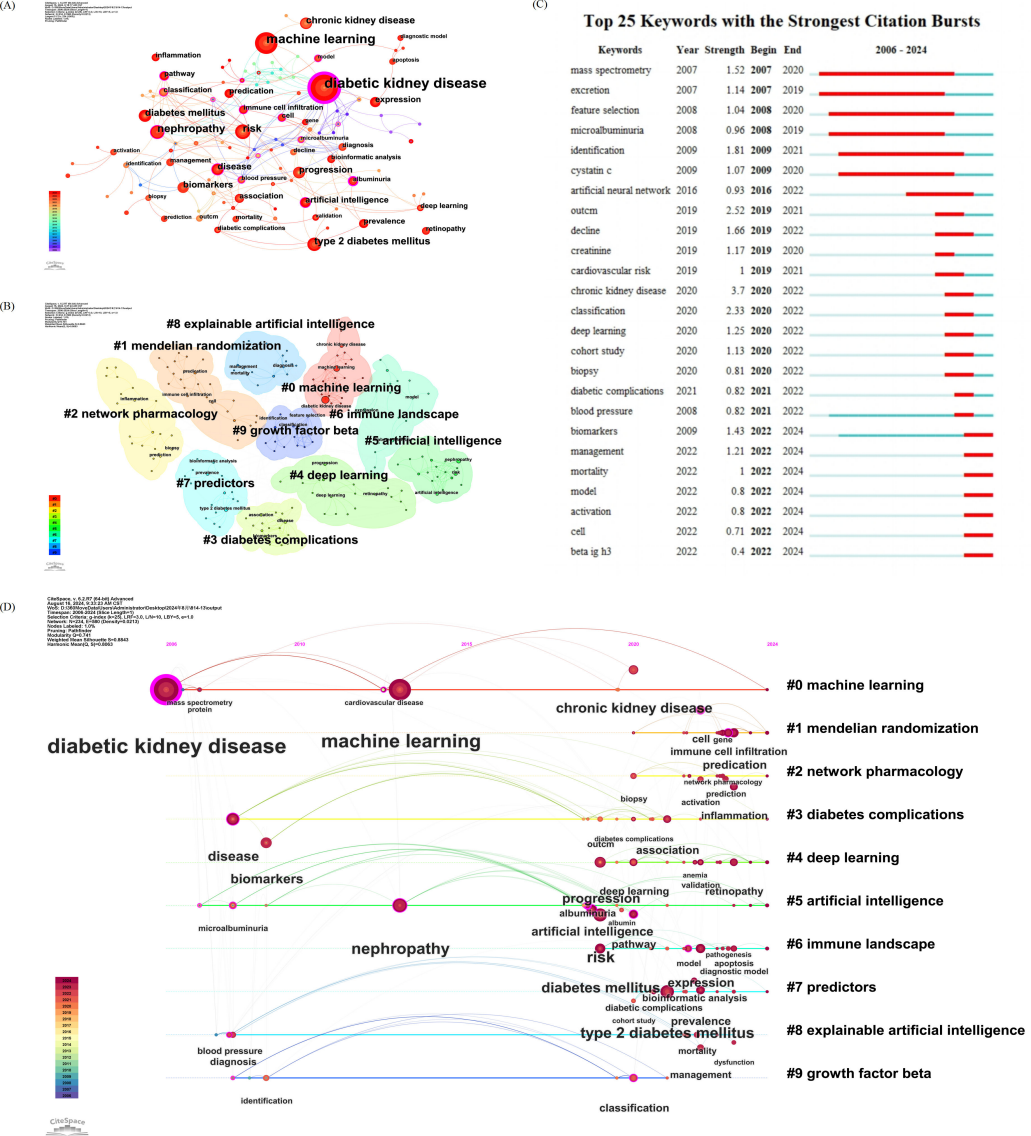
Co-occurrence analysis of keywords in bibliometric studies: (A) keyword co-occurrence network, (B) keyword clustering, (C) keywords with the strongest citation bursts, and (D) timeline of keyword trends in artificial intelligence research on diabetic kidney disease from 2006 to 2024.

Analysis of keyword co-occurrence and burst terms revealed distinct phases in the thematic development of the field. During the early period (2006‐2012), research was focused primarily on pathology, biomarker identification, and proteomic analysis, often using conventional statistical tools. Between 2013 and 2018, machine learning began to emerge as a prominent analytical method, with keywords such as “support vector machine” and “feature selection” gaining prominence. From 2019 onward, deep learning became a dominant theme, as reflected by the increasing frequency of terms such as “convolutional neural network,” “risk prediction,” and “decision support system.” Thematic clustering and citation bursts also indicated a growing interest in explainability, model integration, and individualized risk stratification, marking a shift toward clinical application and interpretability (Figure 3A-D).

### Collaboration Networks Among Authors and Institutions

Coauthorship network visualization demonstrated that the field remains highly fragmented, with a large number of small, loosely connected research groups. The most central nodes in the institutional network were located in China, the United States, and Singapore, reflecting both productivity and cross-institutional engagement. Although multicenter projects were occasionally identified, most AI models were developed and tested within single-center or regional datasets. Cross-national research, while increasing, often lacked shared validation protocols or harmonized data structures, limiting direct comparisons and large-scale model generalizability.

### Model Validation, Explainability, and Translational Readiness

A review of the included studies showed that only a limited proportion of AI models underwent external validation using independent cohorts. Most models were based on retrospective data from a single institution or health system, with internal cross-validation as the primary method of evaluation. Very few studies implemented explainability frameworks such as SHAP or LIME, and even fewer offered insights into how model outputs could be integrated into clinical decision-making processes. Notable exceptions included studies that incorporated prospective testing or demonstrated integration with electronic health records, although these remained rare. DeepMind’s acute kidney injury prediction system, while not DKD-specific, was often cited as a prototype for nephrology-focused AI applications [5]. Similarly, Sumit’s [6] deep learning–based model for chronic kidney disease risk prediction represented an example of real-world implementation relevant to diabetic populations. However, the lack of consistent attention to explainability, real-time integration, and regulatory considerations suggests that most AI-DKD research remains in a pretranslational stage.

## Discussion

### Principal Findings

This bibliometric and thematic analysis presents a comprehensive overview of research trends, international collaborations, and translational depth in the application of AI to DKD from 2006 to 2024. The temporal trend reveals a slow developmental phase lasting more than a decade, followed by a surge in research activity from 2019 onward. This acceleration corresponds with the broader adoption of AI in medicine and the urgent need for precision tools to combat the rising global burden of diabetes-related complications.

China and the United States have emerged as the primary contributors to this field, with China leading in publication quantity and institutional productivity. However, the dominance of single-country studies and weak international collaboration networks suggests a lack of unified global efforts in AI-DKD research. While some cross-border cooperation exists, it has not yet reached the level necessary to support large-scale model generalization or multiethnic validation. Future research should prioritize open data sharing, transnational model calibration, and harmonized validation protocols to promote reproducibility and clinical readiness across diverse populations.

Keyword analysis and thematic clustering indicate a clear evolution in research focus. Early studies emphasized molecular and pathological mechanisms of DKD, typically using traditional regression models or biomarker discovery tools. From 2015 onward, a shift occurred toward applying machine learning algorithms to structured clinical data, including risk prediction and feature selection. Since 2019, the field has seen a rapid proliferation of deep learning–based applications, especially convolutional neural networks for imaging and time-series data analysis. However, the transition from computational innovation to clinical implementation remains incomplete. Most studies prioritize model development and internal validation, while relatively few undertake real-world testing or prospective evaluation.

One major limitation identified is the scarcity of externally validated and clinically integrated AI models. Despite rapid algorithmic progress, few studies reach the level of clinical translation demonstrated by landmark systems such as DeepMind’s acute kidney injury prediction algorithm, which was prospectively validated and tested in hospital settings [5]. Similarly, the work by Sumit [6], which developed and validated a deep learning model for predicting chronic kidney disease progression, represents an exemplar of real-world application. These examples underscore the importance of incorporating prospective design, external datasets, and health system integration early in the research pipeline to ensure that AI tools can transition beyond proof-of-concept stages.

Moreover, the “black box” nature of many AI models presents a significant barrier to clinical trust and regulatory approval. Although explainable artificial intelligence methods such as SHAP and LIME have been proposed and applied in other medical domains, they are seldom used in DKD-related research. This gap not only limits interpretability but also hinders integration into clinical workflows where explainability is essential for physician adoption and patient safety. The increasing interest in interpretable models and hybrid systems—combining clinical rules with machine learning outputs—may offer a promising path forward.

Another noteworthy observation is the underrepresentation of research from low- and middle-income countries, apart from China and India. Given the global prevalence of diabetes and its complications, this imbalance may reflect disparities in AI infrastructure, research funding, and access to large-scale clinical data. Efforts to democratize AI research—such as open-access datasets, international consortia, and capacity-building initiatives—are critical to avoid reinforcing health inequities through algorithmic bias.

### Limitations and Future Work

This study also has limitations. The analysis was based solely on the Web of Science database, which, while comprehensive, may omit relevant studies indexed elsewhere, such as in Scopus or PubMed. The decision to focus on English-language articles may have further excluded important regional research. Additionally, bibliometric tools such as CiteSpace and VOSviewer, while effective in mapping research landscapes, cannot capture the full context or nuance of each study’s methodological rigor or clinical relevance. Therefore, the qualitative thematic analysis presented here serves as a complementary lens, but further domain-specific review is warranted to assess clinical impact.

In conclusion, the field of AI in DKD is rapidly expanding, with increasing interest from diverse geographic regions and institutions. However, the translation of AI models into clinical nephrology practice remains limited. Future research should emphasize multicenter collaboration, external validation, and interpretability to close the gap between computational innovation and real-world impact. A systematic shift toward transparent, validated, and context-aware AI systems will be essential to unlock the full potential of AI in the management of DKD.

### Conclusions

This study provides a comprehensive and multidimensional analysis of the research landscape at the intersection of AI and DKD. Through bibliometric visualization and thematic synthesis, we demonstrate that although the field has experienced substantial growth in recent years—particularly with the application of deep learning technologies—the clinical translation of these innovations remains in its infancy. Most current research is confined to retrospective model development with limited external validation and minimal integration into real-world nephrology practice.

To advance the field, future efforts must prioritize methodological transparency, external validation using diverse populations, and the incorporation of explainable AI frameworks. Strengthening international collaboration and establishing multicenter consortia will be crucial for ensuring reproducibility and promoting equitable access to AI tools across health care settings. Additionally, regulatory and ethical considerations should be proactively addressed to support the safe deployment of AI in clinical decision-making.

In summary, while the promise of AI in DKD is evident, realizing its full potential will require a deliberate transition from algorithmic development to clinically meaningful, patient-centered applications. Bridging this translational gap is not only a technical challenge but also an opportunity to reshape chronic disease management in the era of intelligent medicine.

## Supplementary material

10.2196/72616 Multimedia Appendix 1 Raw bibliometric data exported from the Web of Science Core Collection (CSV format, retrieved May 2024).

10.2196/72616 Multimedia Appendix 2 Analysis scripts and configuration settings used in CiteSpace (version 6.2.R6) and VOSviewer (version 1.6.19), provided in TXT and VOS formats.

## Abbreviations

AI: artificial intelligence
DKD: diabetic kidney disease
LIME: Local Interpretable Model-Agnostic Explanations
PRISMA: Preferred Reporting Items for Systematic Reviews and Meta-Analyses
SHAP: Shapley Additive Explanations
