# Supplementary material for: Artificial Intelligence in Diabetic Kidney Disease Research: Bibliometric Analysis From 2006 to 2024
Source: JMIR Diabetes. 2026 Jan 9;11:e72616. doi: 10.2196/72616 (PMC12786635; doi:10.2196/72616)
Supplement: Multimedia Appendix 1 [file diabetes-v11-e72616-s001.pdf]

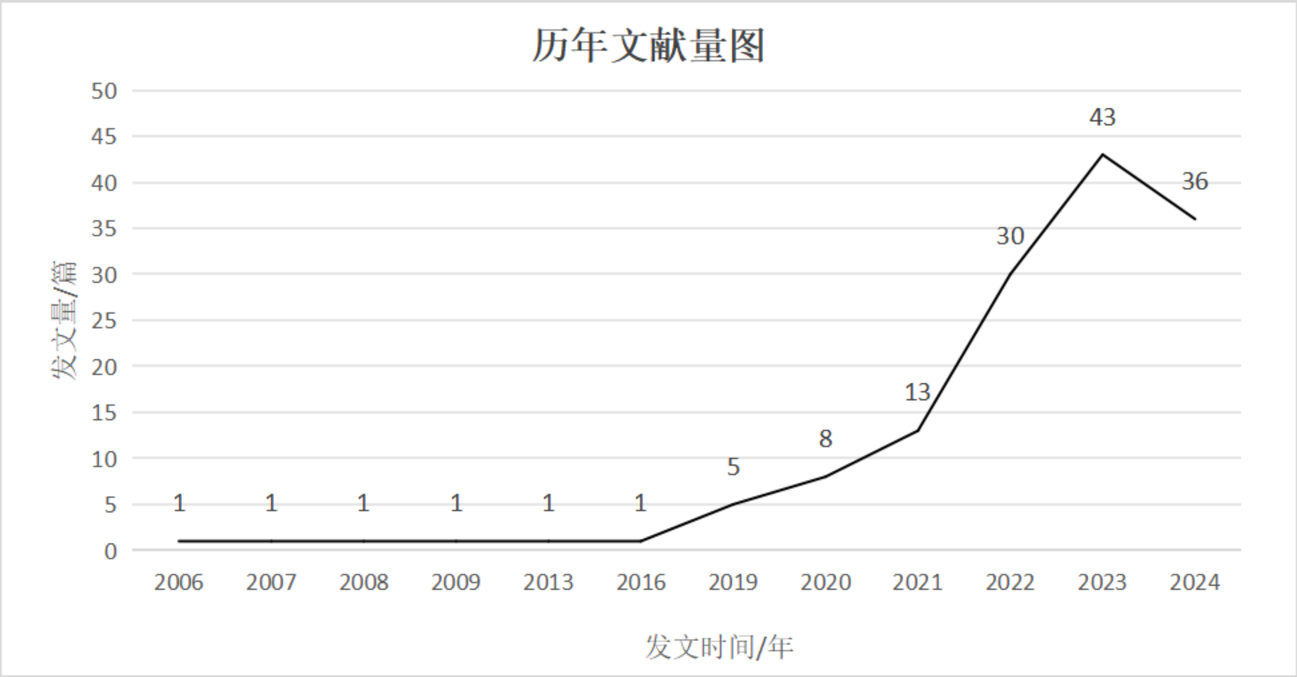

历年文献量图
发文量/篇
50
45
40
35
30
25
20
15
10
5
0
1
1
1
1
1
1
5
8
13
30
43
36
2006
2007
2008
2009
2013
2016
2019
2020
2021
2022
2023
2024
发文时间/年

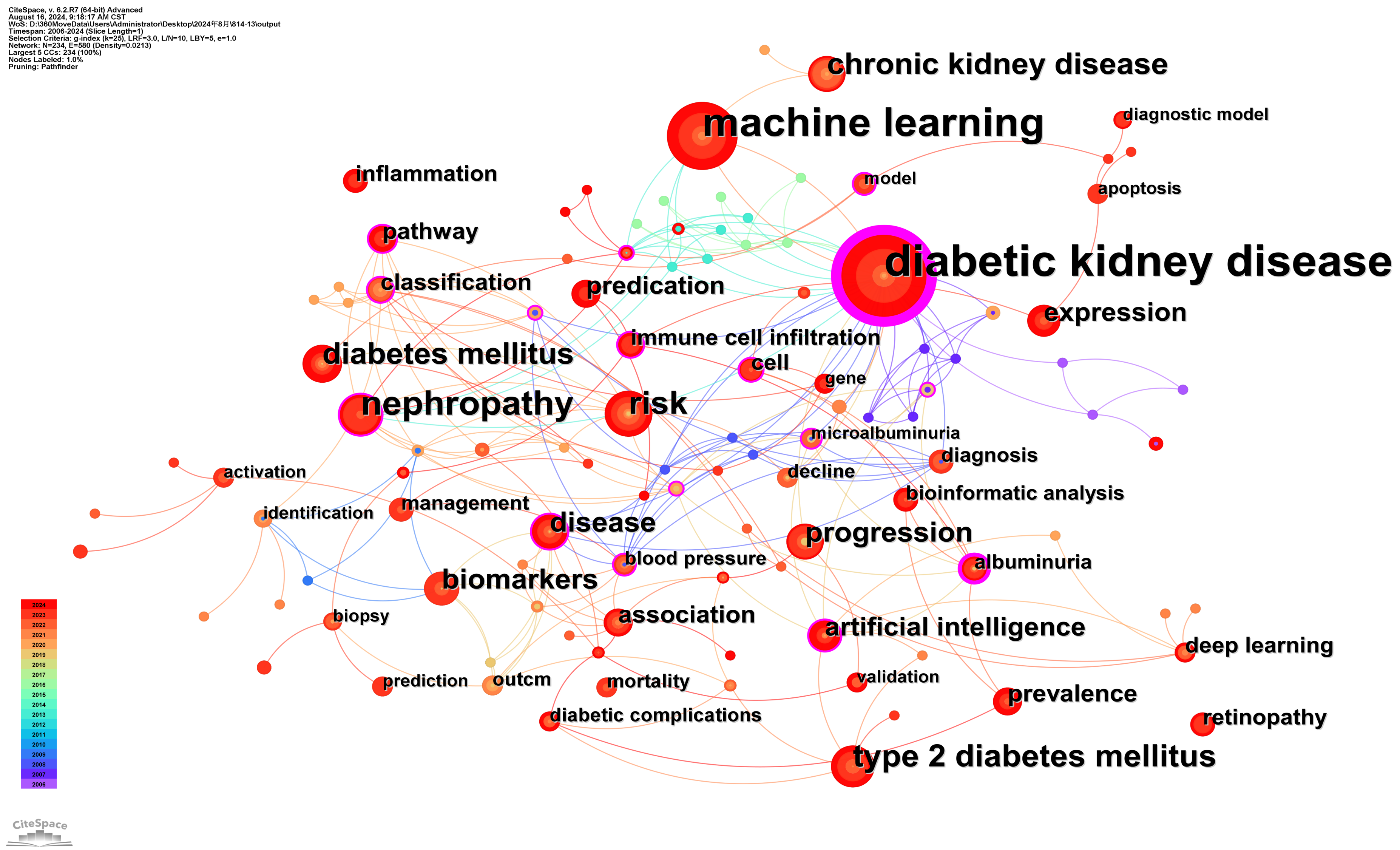
CiteSpace, v. 6.2.R7 (64-bit) Advanced
August 16, 2024, 9:18:17 AM CST
WoS: D:\360MoveData\Users\Administrator\Desktop\2024年8月\814-13\output
Timespan: 2006-2024 (Slice Length=1)
Selection Criteria: g-index (k=25), LRF=3.0, L/N=10, LBY=5, e=1.0
Network: N=234, E=580 (Density=0.0213)
Largest 5 CCs: 234 (100%)
Nodes Labeled: 1.0%
Pruning: Pathfinder
chronic kidney disease
machine learning
diagnostic model
inflammation
model
apoptosis
pathway
diabetic kidney disease
classification
predication
expression
immune cell infiltration
diabetes mellitus
cell
gene
nephropathy
risk
microalbuminuria
diagnosis
activation
decline
bioinformatic analysis
identification
management
disease
progression
blood pressure
albuminuria
biomarkers
association
biopsy
artificial intelligence
deep learning
validation
prediction
outcm
mortality
prevalence
retinopathy
diabetic complications
type 2 diabetes mellitus
2024
2023
2022
2021
2020
2019
2018
2017
2016
2015
2014
2013
2012
2011
2010
2009
2008
2007
2006
CiteSpace

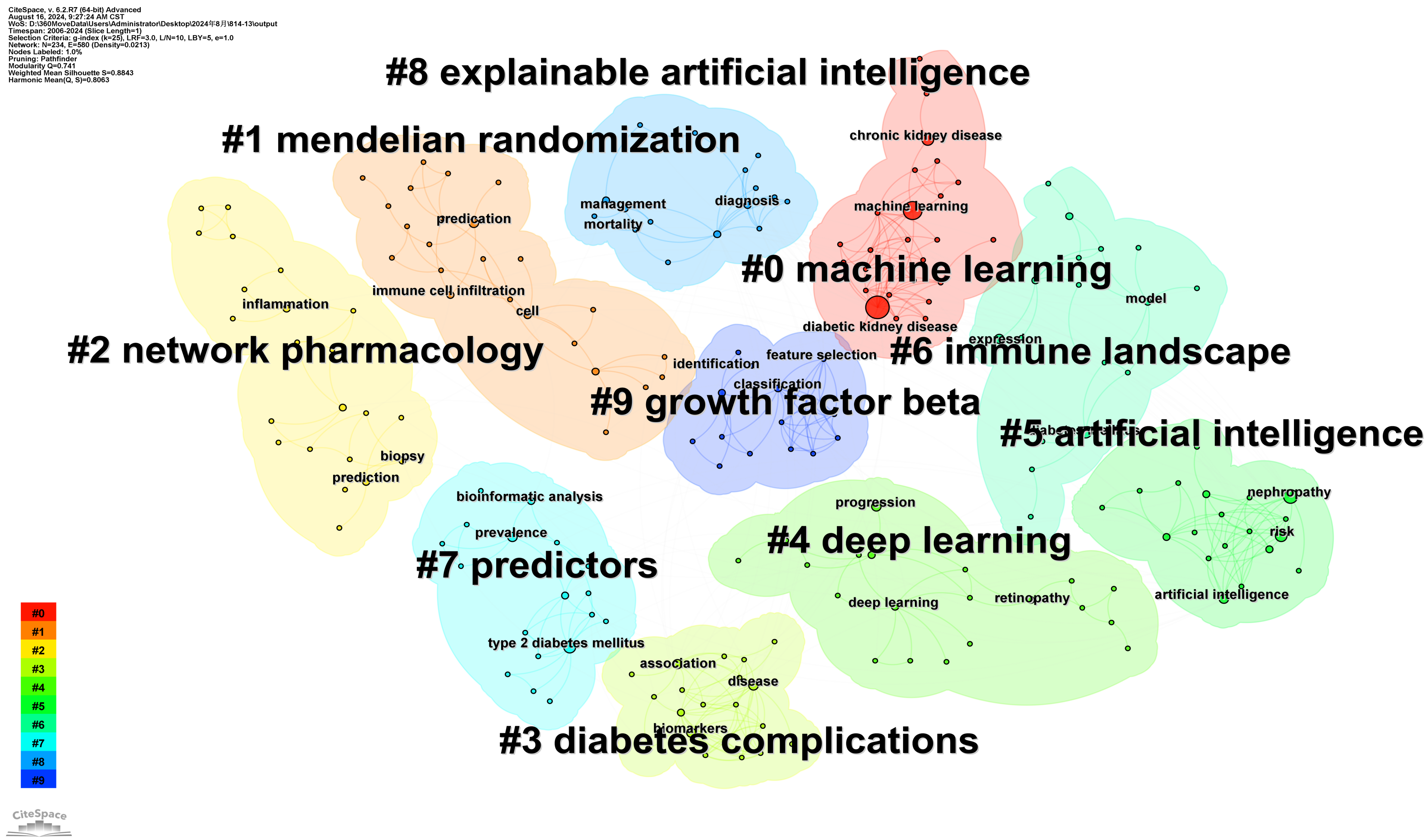

CiteSpace, v. 6.2.R7 (64-bit) Advanced
August 16, 2024, 9:27:24 AM CST
WoS: D:\360MoveData\Users\Administrator\Desktop\2024年8月\814-13\output
Timespan: 2006-2024 (Slice Length=1)
Selection Criteria: g-index (k=25), LRF=3.0, L/N=10, LBY=5, e=1.0
Network: N=234, E=580 (Density=0.0213)
Nodes Labeled: 1.0%
Pruning: Pathfinder
Modularity Q=0.741
Weighted Mean Silhouette S=0.8843
Harmonic Mean(Q, S)=0.8063
#8 explainable artificial intelligence
#1 mendelian randomization
chronic kidney disease
management
diagnosis
mortality
predication
machine learning
#0 machine learning
inflammation
immune cell infiltration
model
cell
diabetic kidney disease
expression
#2 network pharmacology
identification
feature selection
#6 immune landscape
classification
#9 growth factor beta
#5 artificial intelligence
biopsy
prediction
nephropathy
bioinformatic analysis
progression
prevalence
risk
#4 deep learning
#7 predictors
deep learning
retinopathy
artificial intelligence
type 2 diabetes mellitus
association
disease
biomarkers
#3 diabetes complications
#0
#1
#2
#3
#4
#5
#6
#7
#8
#9
CiteSpace

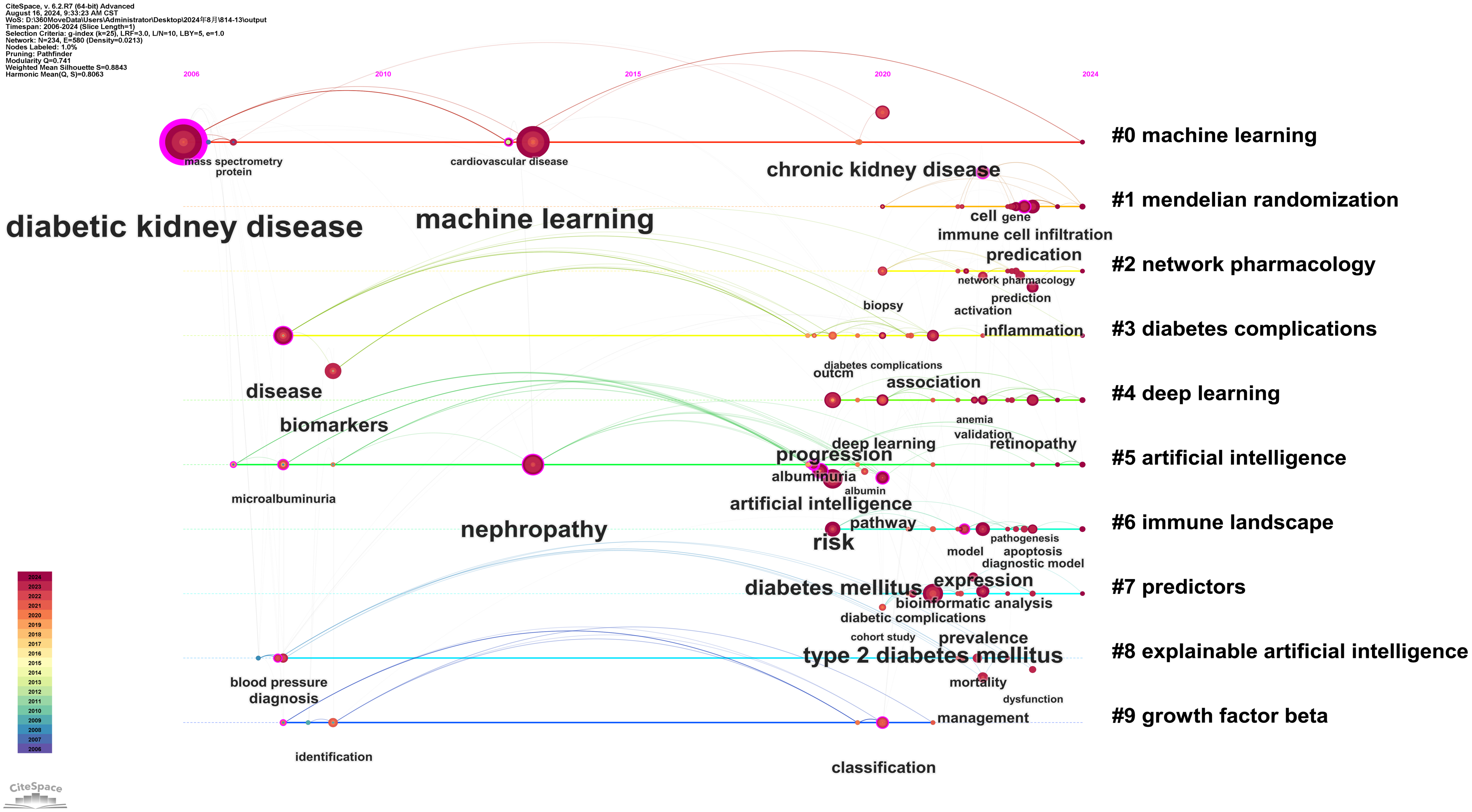

CiteSpace, v. 6.2.R7 (64-bit) Advanced
August 16, 2024, 9:33:23 AM CST
WoS: D:\360MoveData\Users\Administrator\Desktop\2024年8月\814-13\output
Timespan: 2006-2024 (Slice Length=1)
Selection Criteria: g-index (k=25), LRF=3.0, L/N=10, LBY=5, e=1.0
Network: N=234, E=580 (Density=0.0213)
Nodes Labeled: 1.0%
Pruning: Pathfinder
Modularity Q=0.741
Weighted Mean Silhouette S=0.8843
Harmonic Mean(Q, S)=0.8063
2006
2010
2015
2020
2024
#0 machine learning
#1 mendelian randomization
#2 network pharmacology
#3 diabetes complications
#4 deep learning
#5 artificial intelligence
#6 immune landscape
#7 predictors
#8 explainable artificial intelligence
#9 growth factor beta
mass spectrometry
protein
cardiovascular disease
chronic kidney disease
diabetic kidney disease
machine learning
cell
gene
immune cell infiltration
predication
network pharmacology
biopsy
prediction
activation
inflammation
disease
diabetes complications
outcm
association
biomarkers
anemia
validation
deep learning
retinopathy
progression
albuminuria
albumin
microalbuminuria
artificial intelligence
pathway
nephropathy
risk
pathogenesis
model
apoptosis
diagnostic model
expression
diabetes mellitus
bioinformatic analysis
diabetic complications
cohort study
prevalence
type 2 diabetes mellitus
mortality
blood pressure
diagnosis
dysfunction
management
identification
classification
2024
2023
2022
2021
2020
2019
2018
2017
2016
2015
2014
2013
2012
2011
2010
2009
2008
2007
2006
CiteSpace

Top 25 Keywords with the Strongest Citation Bursts

Keywords	Year	Strength	Begin	End	2006 - 2024
mass spectrometry	2007	1.52	**2007**	2020	
excretion	2007	1.14	**2007**	2019	
feature selection	2008	1.04	**2008**	2020	
microalbuminuria	2008	0.96	**2008**	2019	
identification	2009	1.81	**2009**	2021	
cystatin c	2009	1.07	**2009**	2020	
artificial neural network	2016	0.93	**2016**	2022	
outcm	2019	2.52	**2019**	2021	
decline	2019	1.66	**2019**	2022	
creatinine	2019	1.17	**2019**	2020	
cardiovascular risk	2019	1	**2019**	2021	
chronic kidney disease	2020	3.7	**2020**	2022	
classification	2020	2.33	**2020**	2022	
deep learning	2020	1.25	**2020**	2022	
cohort study	2020	1.13	**2020**	2022	
biopsy	2020	0.81	**2020**	2022	
diabetic complications	2021	0.82	**2021**	2022	
blood pressure	2008	0.82	**2021**	2022	
biomarkers	2009	1.43	**2022**	2024	
management	2022	1.21	**2022**	2024	
mortality	2022	1	**2022**	2024	
model	2022	0.8	**2022**	2024	
activation	2022	0.8	**2022**	2024	
cell	2022	0.71	**2022**	2024	
beta ig h3	2022	0.4	**2022**	2024	

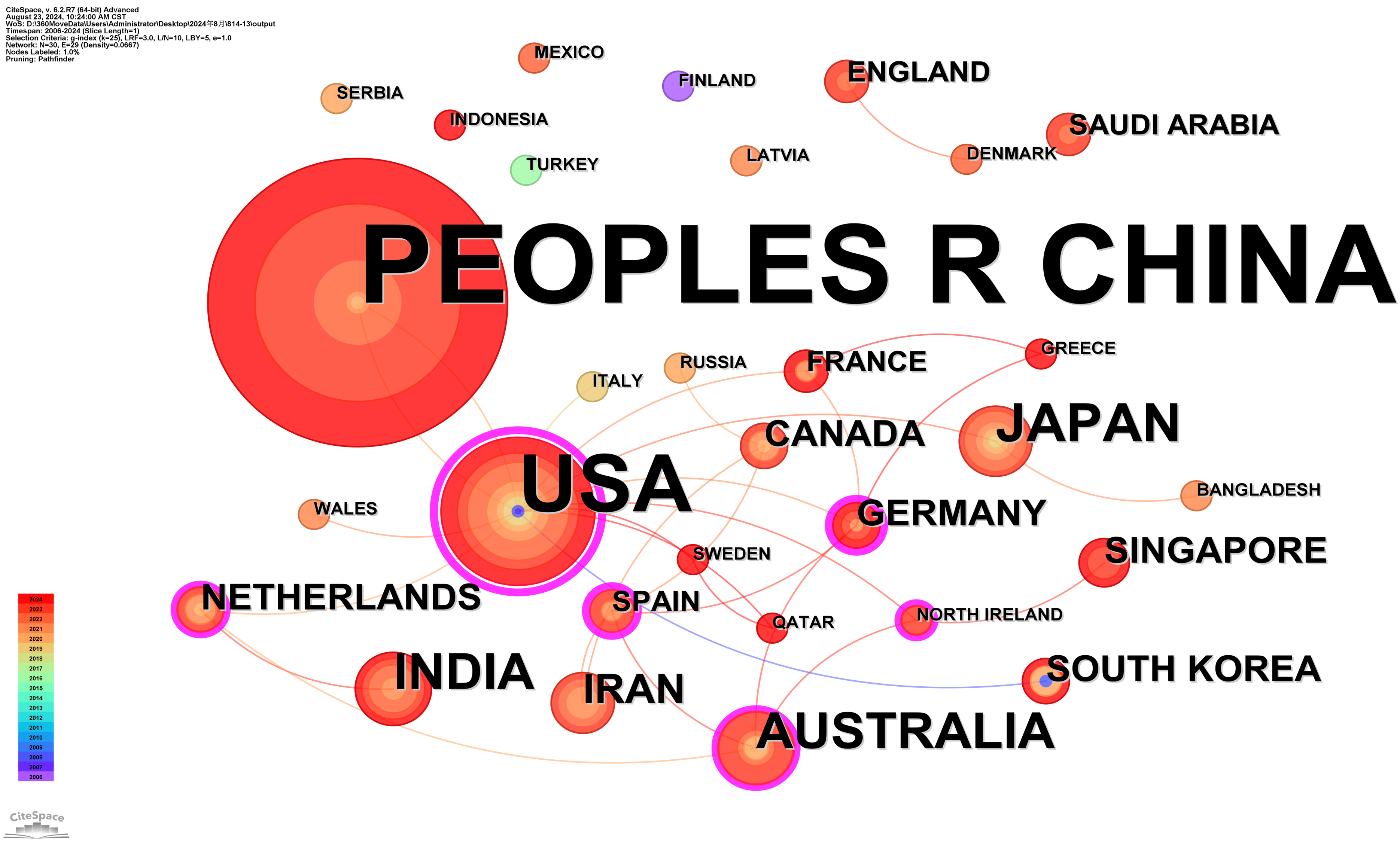
CiteSpace, v. 6.2.R7 (64-bit) Advanced
August 23, 2024, 10:24:00 AM CST
WoS: D:\360MoveData\Users\Administrator\Desktop\2024年8月\814-13\output
Timespan: 2006-2024 (Slice Length=1)
Selection Criteria: g-index (k=25), LRF=3.0, L/N=10, LBY=5, e=1.0
Network: N=30, E=29 (Density=0.0667)
Nodes Labeled: 1.0%
Pruning: Pathfinder
MEXICO
FINLAND
ENGLAND
SERBIA
INDONESIA
SAUDI ARABIA
TURKEY
LATVIA
DENMARK
PEOPLES R CHINA
GREECE
RUSSIA
FRANCE
ITALY
JAPAN
CANADA
USA
BANGLADESH
WALES
GERMANY
SINGAPORE
SWEDEN
NETHERLANDS
SPAIN
QATAR
NORTH IRELAND
INDIA
IRAN
SOUTH KOREA
AUSTRALIA
2024
2023
2022
2021
2020
2019
2018
2017
2016
2015
2014
2013
2012
2011
2010
2009
2008
2007
2006
CiteSpace

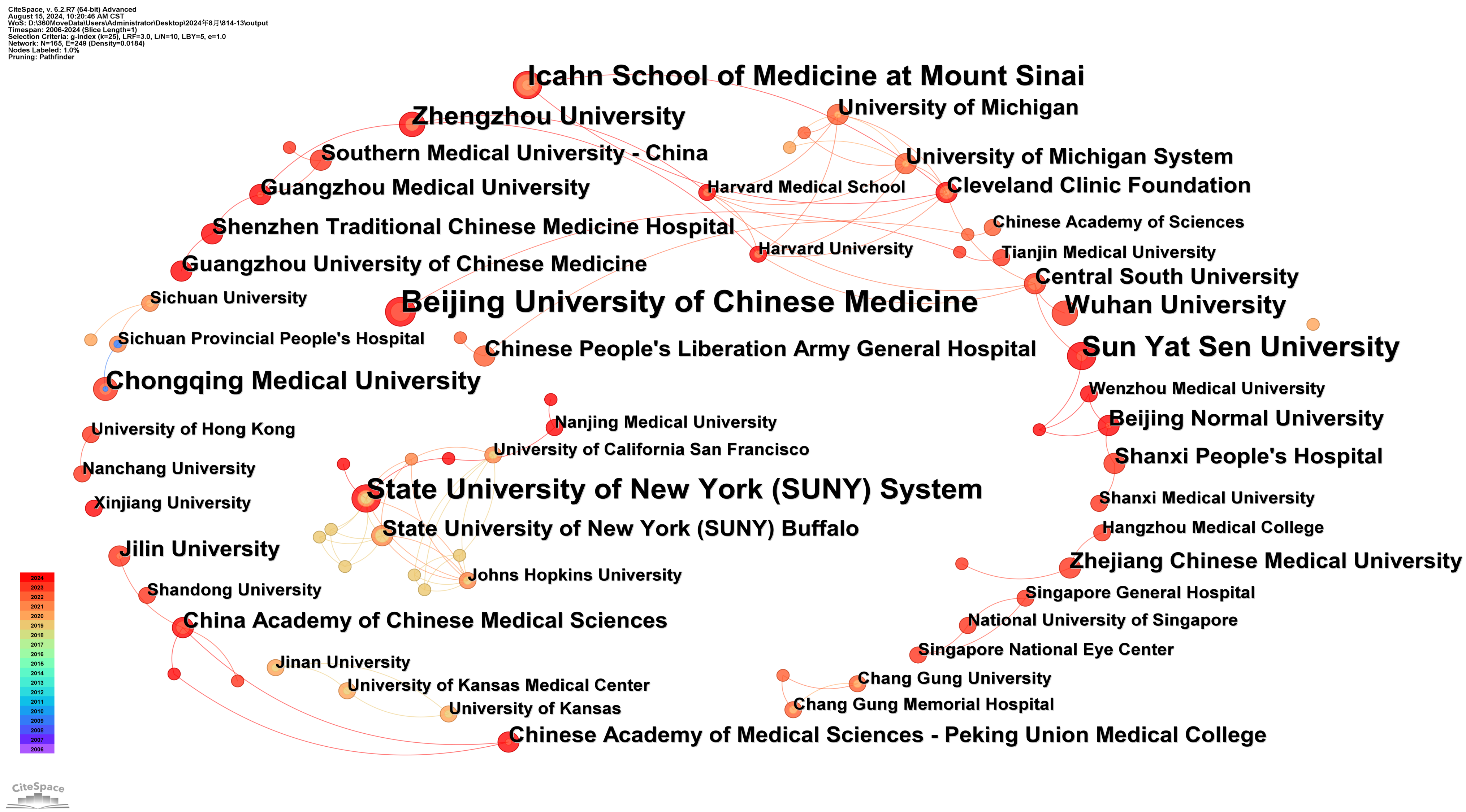
CiteSpace, v. 6.2.R7 (64-bit) Advanced
August 15, 2024, 10:20:46 AM CST
WoS: D:\360MoveData\Users\Administrator\Desktop\2024年8月\814-13\output
Timespan: 2006-2024 (Slice Length=1)
Selection Criteria: g-index (k=25), LRF=3.0, L/N=10, LBY=5, e=1.0
Network: N=165, E=249 (Density=0.0184)
Nodes Labeled: 1.0%
Pruning: Pathfinder
Icahn School of Medicine at Mount Sinai
University of Michigan
Zhengzhou University
Southern Medical University - China
University of Michigan System
Harvard Medical School
Cleveland Clinic Foundation
Guangzhou Medical University
Chinese Academy of Sciences
Shenzhen Traditional Chinese Medicine Hospital
Harvard University
Tianjin Medical University
Guangzhou University of Chinese Medicine
Central South University
Sichuan University
Beijing University of Chinese Medicine
Wuhan University
Sichuan Provincial People's Hospital
Sun Yat Sen University
Chinese People's Liberation Army General Hospital
Chongqing Medical University
Wenzhou Medical University
Beijing Normal University
Nanjing Medical University
University of Hong Kong
University of California San Francisco
Shanxi People's Hospital
Nanchang University
State University of New York (SUNY) System
Xinjiang University
Shanxi Medical University
State University of New York (SUNY) Buffalo
Hangzhou Medical College
Jilin University
Zhejiang Chinese Medical University
Johns Hopkins University
Shandong University
Singapore General Hospital
China Academy of Chinese Medical Sciences
National University of Singapore
Singapore National Eye Center
Jinan University
Chang Gung University
University of Kansas Medical Center
Chang Gung Memorial Hospital
University of Kansas
Chinese Academy of Medical Sciences - Peking Union Medical College
2024
2023
2022
2021
2020
2019
2018
2017
2016
2015
2014
2013
2012
2011
2010
2009
2008
2007
2006
CiteSpace

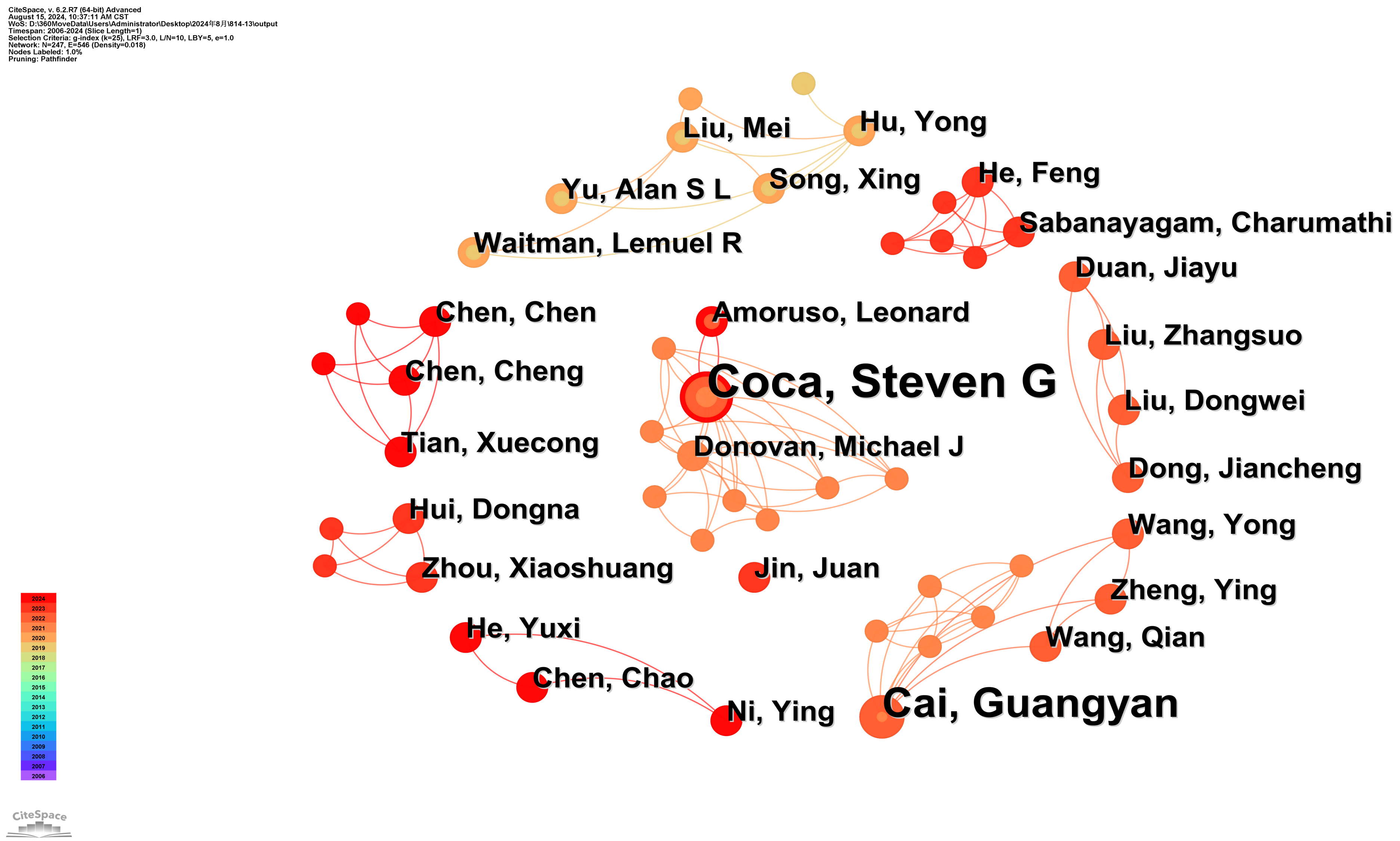
CiteSpace, v. 6.2.R7 (64-bit) Advanced
August 15, 2024, 10:37:11 AM CST
WoS: D:\360MoveData\Users\Administrator\Desktop\2024年8月\814-13\output
Timespan: 2006-2024 (Slice Length=1)
Selection Criteria: g-index (k=25), LRF=3.0, L/N=10, LBY=5, e=1.0
Network: N=247, E=546 (Density=0.018)
Nodes Labeled: 1.0%
Pruning: Pathfinder
Liu, Mei
Hu, Yong
Yu, Alan S L
Song, Xing
He, Feng
Sabanayagam, Charumathi
Waitman, Lemuel R
Duan, Jiayu
Chen, Chen
Amoruso, Leonard
Liu, Zhangsuo
Chen, Cheng
Coca, Steven G
Liu, Dongwei
Tian, Xuecong
Donovan, Michael J
Dong, Jiancheng
Hui, Dongna
Wang, Yong
Zhou, Xiaoshuang
Jin, Juan
Zheng, Ying
He, Yuxi
Wang, Qian
Chen, Chao
Ni, Ying
Cai, Guangyan
2024
2023
2022
2021
2020
2019
2018
2017
2016
2015
2014
2013
2012
2011
2010
2009
2008
2007
2006
CiteSpace
